# Unsuspected radiolucent partial denture impaction in the sigmoid colon mimicking malignancy

**DOI:** 10.1259/bjrcr.20160074

**Published:** 2016-12-19

**Authors:** Makabongwe Tshuma, Vamsi Velchuru, Geoffrey Andrew Waters

**Affiliations:** ^1^Radiology and Pathology, Norfolk and Norwich University Hospital, Norwich, England, UK; ^2^General Surgery, James Paget University Hospital, Great Yarmouth, England, UK

## Abstract

We report the case of a 79-year-old male who presented with altered bowel habit. Subsequent endoscopy and radiological investigations suggested the diagnosis of a likely sigmoid colon malignancy. Surgery was performed to remove the suspected tumour and post-surgical histology showed only inflammatory change in an area of underlying sigmoid diverticulosis with an associated impacted dental prosthesis.

Accidental ingestion of foreign bodies in adults is not uncommon; however, the majority pass through the alimentary canal without incident.^1^ The ones that do not pass usually present as trapped foreign bodies within the pharynx or oesophagus with the common culprit being mainly bones and meat boluses.^1^ There are numerous factors that can contribute to the risk of swallowed foreign body in the adult population including alcoholism, old age, mental health problems and history of stroke or neuromuscular degenerative conditions.

Within the elderly population the aspiration/ingestion of dental prosthesis is more common.^2^ The National Health and Nutrition Examination Survey (NHANES III) found that one in every five persons aged between 18 and 74 years has full or partial dentures.^3^ However, as a result of the size and rigidity of dental plates, most patients who have accidentally ingested present early with upper gastrointestinal tract symptoms. Occasionally, these pass through to the stomach and into the small and large bowel where they can either pass through without incident or become impacted and cause an obstruction and/or perforation.^2^

Plain radiographs are the mainstay initial investigation for swallowed foreign bodies, especially dental material, as most dental prostheses are radiopaque.^4^ The base material used to make most removable dental prostheses, polymethylmethacrylate (PMMA), is radiolucent;^5,6^ this creates a diagnostic challenge following accidental ingestion as radiological tracking of the ingested denture/partial denture becomes difficult.

We present a case where impaction of the partial denture within the sigmoid colon mimicked carcinoma on endoscopy and subsequently on radiological evaluation.

## Case report

A 79-year-old male was referred to the surgical outpatient department with a 2-month history of altered bowel habit. Abdominal examination and digital rectal examination in the outpatient clinic were unremarkable.

**Figure 1.**
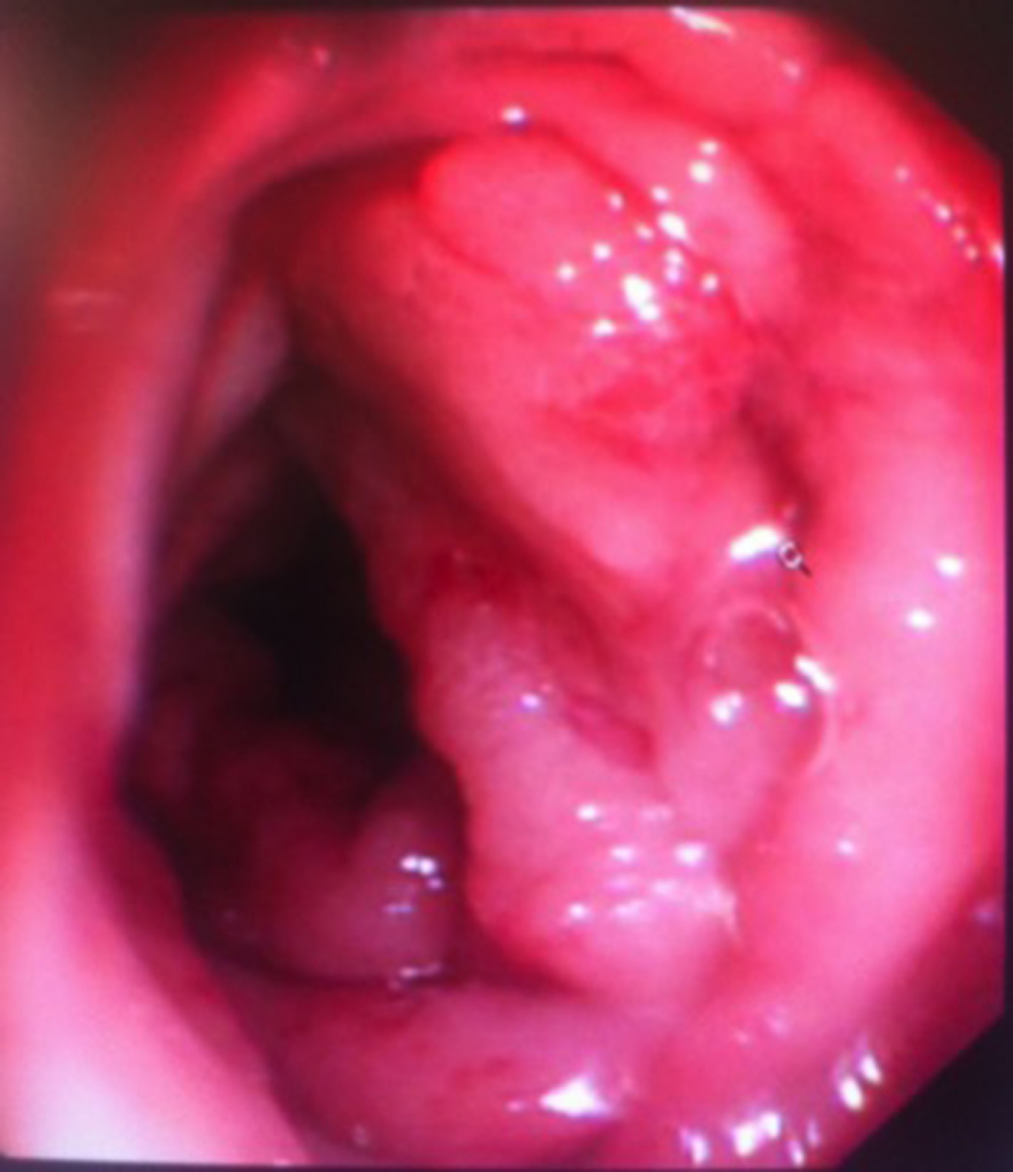
Photograph showing the malignant appearing stricturing sigmoid lesion seen at 25 cm from anal verge taken during colonoscopy.

The laboratory studies, full blood count and biochemistry were normal. A colonoscopy study identified four abnormal lesions, two of which were colonic subcentimetre polyps. More distally in the mid-rectum, at 15 cm from the anal verge, there was a sizeable, villous polypoidal lesion, which was proved on biopsy to be a tubulovillous adenoma (TVA) showing low-grade dysplasia. More proximally in the distal sigmoid colon at 25 cm there was a segment of a malignant-looking lesion associated with a stricture (Figure 1). However, biopsy samples from this region showed hyperplastic colonic mucosa showing lamina propria fibrosis and mild chronic active inflammation with separate pieces of granulation tissue and ulcer slough but no evidence of malignancy.

**Figure 2.**
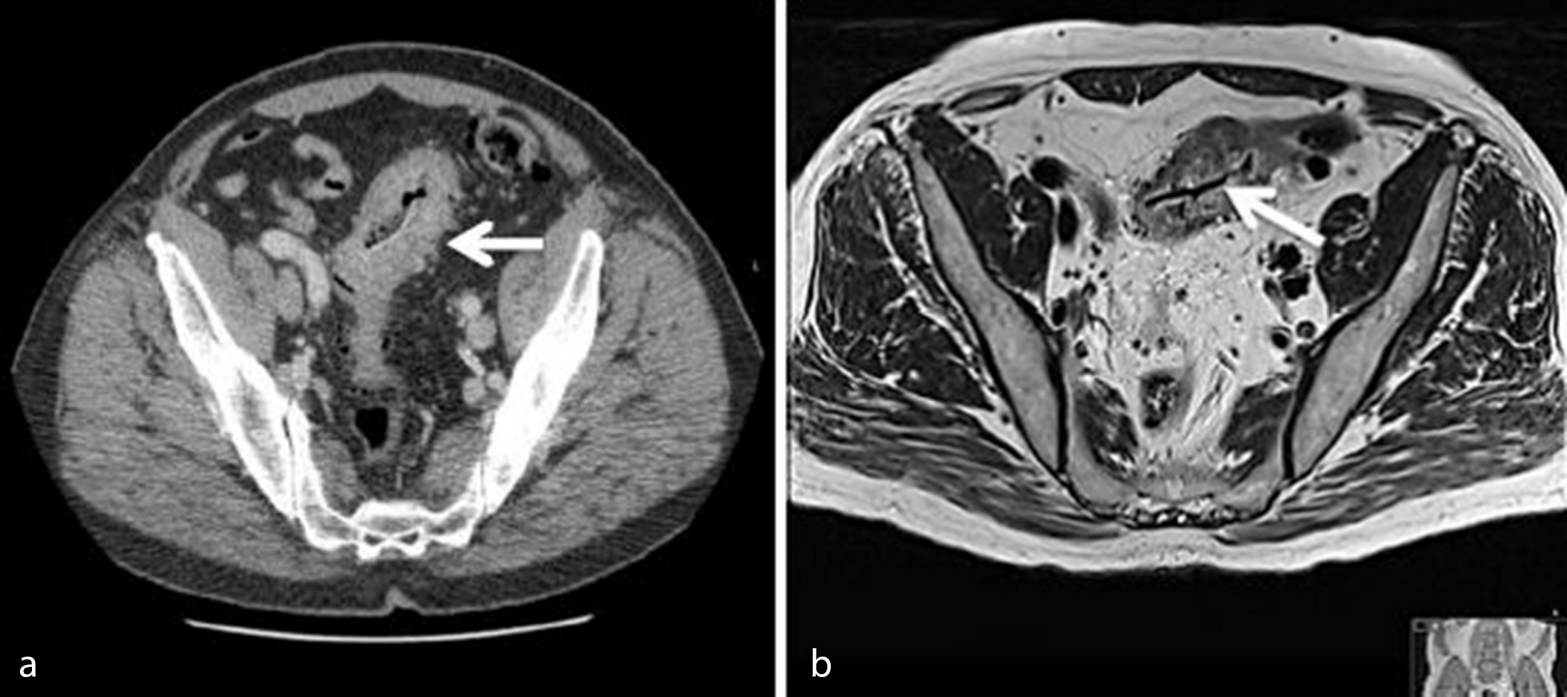
(a) CT axial slice through the pelvis showing the thickened distal sigmoid colon with multiple diverticulae (white arrow). The linear hyperdense line on the intraluminal surface represents the poorly demarcated dental prosthesis. (b) MRI axial slice corresponding to the CT image showing an intraluminal low signal, well-defined linear structure corresponding to the dental prosthesis.

As per local protocol for malignant-appearing lesions on endoscopy, a staging CT scan was arranged on the basis of the colonoscopy appearances to facilitate the cancer pathway. This demonstrated, at the site of the presumed malignancy in the distal sigmoid colon, a 7 cm segment of circumferential thickening on a background of diverticulosis (Figure 2a). This was associated with a few subcentimetre locoregional and inferior mesenteric lymph nodes. There was no suggestion of malignancy elsewhere; so the proposed CT staging was T2/3 N1 M0.

**Figure 3.**
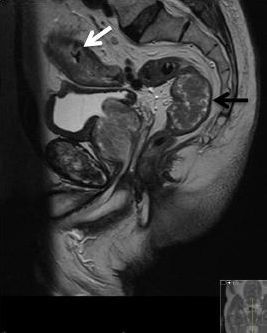
Sagittal *T*_2_ sequences showing part of the dental plate (white arrow) in the thickened distal sigmoid colon and the rectal tubulovillous adenoma (black arrow).

Although the 5 cm rectal TVA could explain the patient’s symptoms it was felt at the multidisciplinary team discussion that either lesion could be responsible for the patient’s symptoms. Despite the lack of malignant cells from the biopsy samples the surgical team were still concerned about a potential sigmoid colon malignancy from the endoscopy and CT appearances. It was felt that the colonoscopy sampling was not truly representative of the malignant appearing sigmoid colon lesion seen at colonoscopy and CT. An anterior resection was planned for the patient to remove both the TVA and presumed sigmoid colon malignancy. An MRI scan of the rectum was performed in order to preoperatively assess the rectal polyp further. The sigmoid tumour was incidentally included in the MRI scan field owing to proximity to the rectal lesion (Figure 3); however, this did not add any diagnostic value preoperatively.

**Figure 4.**
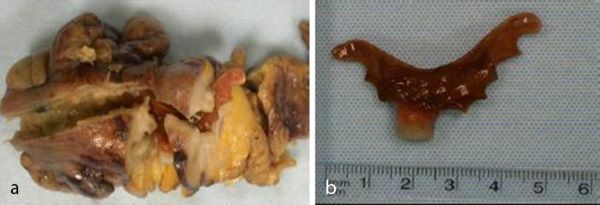
(a) Histological specimen showing the section of the distal sigmoid colon presumed to be malignant and the edges of the dental plate. Diverticular out-pouchings can also been seen in this specimen. (b) The recovered partial denture with a single tooth measuring 5 cm in longest length with sharp jagged edges that presumably contributed to its impaction.

The patient underwent an uncomplicated laparoscopic-assisted anterior resection with defunctioning loop colostomy. Post-surgical histology confirmed that the rectal polyp was a low-grade TVA. However, histological assessment of the presumed distal sigmoid cancer at 25 cm demonstrated mucosal ulceration with underlying fibrosis and diverticulosis. The ulceration was associated with a 5 cm jagged edged intraluminal dental plate containing a single front upper incisor prosthetic tooth found impacted at this site, with no metallic components (Figure 4a,b). The dental prosthesis was so impacted that endoscopic retrieval would be unlikely to have been successful. No malignancy was demonstrated in the post-surgical specimen. There were a few enlarged local lymph nodes present in the specimen, which were all benign. The patient had a good post-surgical recovery and went on to have an uncomplicated reversal of defunctioning colostomy several months later.

**Figure 5.**
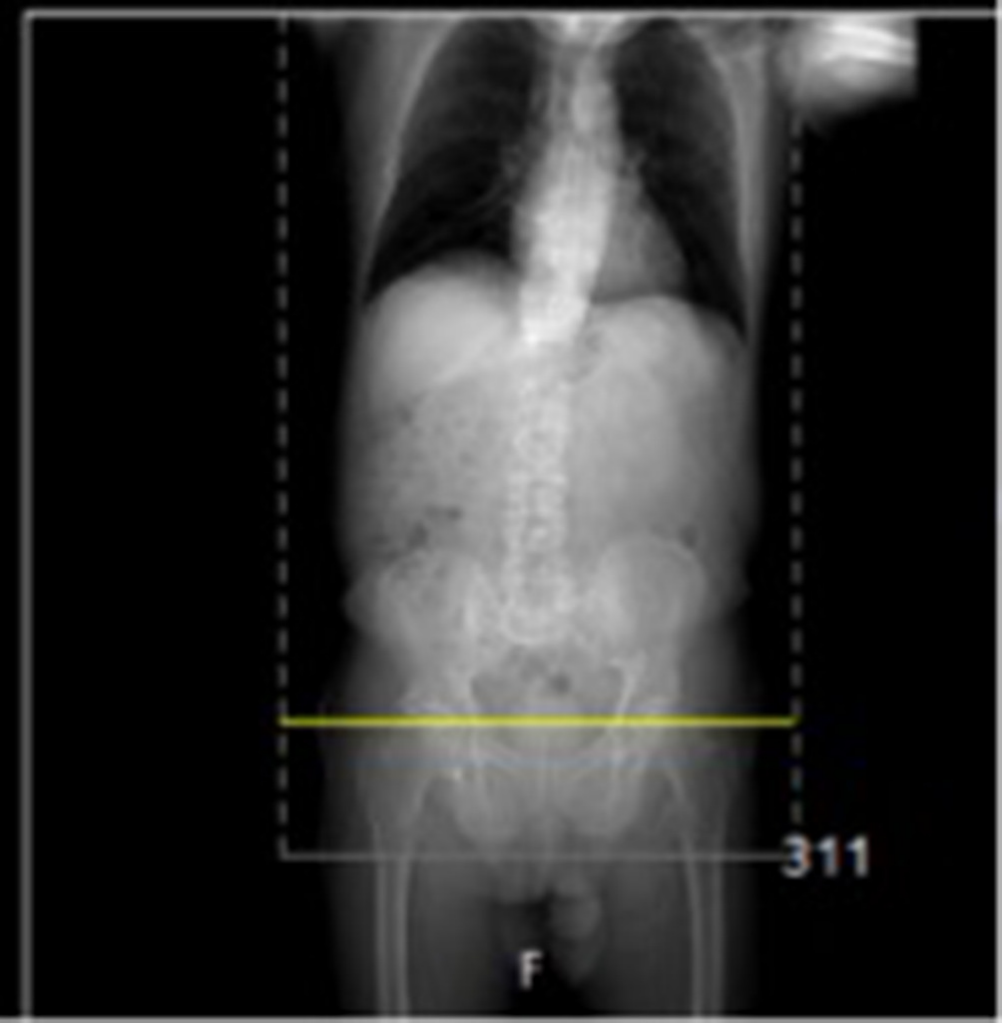
The CT scout view shows no radiopaque foreign body.

Retrospective review of the patient’s previous imaging revealed that the patient had attended the accident and emergency department 2 years earlier, having accidentally swallowed “false teeth” during a meal. An abdominal plain radiograph had been taken at the time, which did not demonstrate any radiopaque foreign body. The CT scout view also demonstrates no radiopaque foreign body (Figure 5). The patient denied any symptoms and had been discharged with no further follow-up. Interestingly, the patient claims he has always known that he had not passed the swallowed dental prosthesis.

## Discussion

Most available literature on cases of complications of accidentally swallowed dental prostheses describes acute oesophageal impaction and related complications owing to the size of the prostheses.^7^ However, as in the case patient, more distal colonic impaction of foreign bodies may result in serious late complications, especially in the presence of significant colonic diverticulosis. The prevalence of colonic diverticulosis is 65% from the age of 80 years onwards^8^ and risk of aspiration is also high in this age group. There is a higher risk of impaction associated with dental prostheses owing to their size.

Just as failure of migration on imaging is concerning for obstruction or perforation for swallowed radiopaque materials,^4^ failure to pass through the alimentary canal after several weeks should also raise the possibility of impaction—particularly at an area of narrowing such as the ileocaecal valve or a colonic lesion causing luminal narrowing such as an area of significant diverticular change. In the case reported by Tanrikulu et al,^9^ the swallowed lower denture was radiopaque and surgery was performed when ileocaecal impaction occurred leading to obstructive symptoms. In another case reported by Ghori et al,^1^ sigmoid perforation from the partial denture occurred with a fatal outcome to a young patient despite surgical management.

In this case, it is not entirely clear which lesion would have caused the patient’s symptoms but surgery was necessary in order to alleviate his symptoms. Given the location of the dental prosthesis and the duration of 2 years since ingestion it is likely that the relatively recent bowel symptoms were all owing to a more recent lesion such as the TVA. However, with progression of the sigmoid diverticulosis and associated inflammatory changes future complications would still be possible had surgery not been performed.

The question remains whether all dental prosthesis should have a radiopaque marker to assist with tracking in the event of accidental ingestion/aspiration. Retrospective analysis of the contrast-enhanced CT scan images reveal a thin linear area of slight hyperdensity in the intraluminal region of the sigmoid colon lesion (Figure 2a) that could easily be interpreted as mucosal enhancement in the absence of the history of possible foreign body. This corresponds to the slightly more prominent well-defined T2 low signal, linear intraluminal structure in the same region on the MR images (Figure 2b). The colonic intraluminal contents are generally of low signal on MRI and together with the colonoscopy findings, without the history of swallowed foreign body, the diagnosis of an impacted dental prosthesis could not have been made.

Both CT and MRI did not reveal any metal artefact and the histological specimen did not demonstrate any metal component in keeping with a polymethylmethacrylate dental prosthesis, thereby making accurate imaging diagnosis difficult. Chawla et al describe a characteristic curvilinear or irregular mildly hyperdense structure on unenhanced CT within the oesophagus in their four cases of impacted dentures.^7^ Although this is similar to the CT findings in our case patient, (Figure 2a) the presence of intravenous iodinated contrast would have reduced sensitivity. However, it is useful to know that these radiopaque dental prostheses are mildly hyperdense on unenhanced CT.

In summary, in the absence of radiologic findings, with a clear history of ingestion of a sizeable dental prosthesis, it is advisable to examine further with an oesophagogastric duodenoscopy at presentation, and interval colonoscopy if still not passed to reduce the risk of foreign body-related obstruction, impaction or perforation.^10^

## Learning points

1. Ingested dental prostheses can prove a diagnostic challenge on imaging, particularly in the absence of metal components. There is a role for CT imaging in investigating ingested dental prostheses, particularly if colonic impaction is suspected.
2. In the absence of radiologic findings, with a clear history of ingestion of a sizeable dental prosthesis, it is advisable to examine further with an OGD at presentation, and an interval colonoscopy if not passed to reduce the risk of foreign body-related obstruction or perforation.
3. Radiologists should have a heightened sense of awareness to the possibility of radiolucent foreign bodies and dentures that may get impacted in the presence of significant colonic diverticulosis in the elderly. This may alert them to the finding of subtle linear intraluminal structure on CT and MRI.
4. Chronic sigmoid diverticulitis or significant sigmoid diverticulosis can mimic a malignancy on CT imaging and on endoscopy; so care needs to be taken to avoid making an over diagnosis particularly when biopsy samples are negative for malignancy.

## Consent

Written informed consent was obtained from the patient(s) for publication of this case report, including accompanying images.
